# Acute central retinal artery occlusion with emboli

**DOI:** 10.1002/ccr3.7435

**Published:** 2023-07-05

**Authors:** Argyrios Chronopoulos, Goergios Chatzantonis, James Scott Schutz, Lars‐Olof Hattenbach

**Affiliations:** ^1^ Department of Ophthalmology Hospital of Ludwigshafen am Rhein Ludwigshafen Germany; ^2^ Department of Vascular Surgery Hospital of Ludwigshafen am Rhein Ludwigshafen Germany; ^3^ 1st Clinic of Vascular Surgery Henry Dunant Hospital Center Athens Greece

**Keywords:** central retinal artery occlusion, retinal artery embolism, retinal ischemia, stroke

## Abstract

CRAO is an ophthalmic and medical emergency. This case is a reminder that diagnosis and management of CRAO begins with ophthalmologists but immediately thereafter care involves emergency cardiovascular and neurological similar to cerebral stroke.

Does central retinal artery occlusion (CRAO) warrant emergency evaluation similar to ischemic stroke?

A 79‐year‐old man presented with a painless sudden loss of vision from his right eye upon awakening. Examination of the right eye demonstrated an afferent pupillary defect, prominent cherry red spot, diffuse retinal arterial narrowing, retinal emboli (Figure 1A, Video S1), and delayed arm‐retina filling time (21 s) on fluorescein angiography (Figure 1B). CRAO was diagnosed and emergency cardiovascular and neurological assessment revealed disseminated right internal carotid artery calcification without relevant stenosis, no valvular abnormalities, and previously undiagnosed atrial fibrillation. The patient was put on a double antiplatelet and anticoagulant treatment.

**FIGURE 1:**
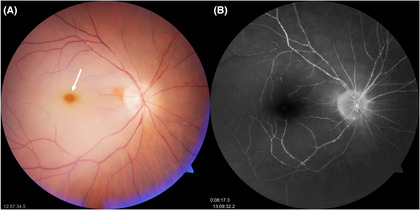
(A) Central retinal artery occlusion with cherry red spot (white arrow) and multiple disseminated small cholesterol and calcific emboli in narrowed retinal arterial branches. (B) fluorescence angiography with flow discontinuities from disseminated retinal arterial emboli.

In cases of CRAO, particularly embolic, emergency systemic evaluation is indicated because such patients are prone to cerebral stroke.^1^ Ultrasound vascular assessment in emergency cases can be readily performed and can provide valuable information as a marker of endothelial vascular dysfunction helping identify individuals at increased cardiovascular risk.^2^

## AUTHOR CONTRIBUTIONS

**Argyrios Chronopoulos:** Conceptualization; data curation; formal analysis; investigation; methodology; project administration; validation; visualization; writing – original draft. **Georgios Chatzantonis:** Formal analysis; investigation; validation. **James Scott Schutz:** Formal analysis; methodology; project administration; supervision; validation; visualization; writing – original draft. **Lars‐Olof Hattenbach:** Resources; supervision; validation; visualization.

## FUNDING INFORMATION

There is no funding to declare.

## CONFLICT OF INTEREST STATEMENT

The authors have no conflicts to declare.

## CONSENT

Written informed consent was obtained from the patient to publish this report in accordance with the journal's patient consent policy.

## ETHICAL APPROVAL

Not applicable.

## Supporting information


Video S1:

## Data Availability

The data that support the findings of this study are available on request from the corresponding author.
